# Multivariate Analysis Revealed Ultrasonic-Assisted Extraction Improves Anti-Melanoma Activity of Non-Flavonoid Compounds in Indonesian Brown Algae Ethanol Extract

**DOI:** 10.3390/molecules27217509

**Published:** 2022-11-03

**Authors:** Anggit Listyacahyani Sunarwidhi, Ari Hernawan, Andri Frediansyah, Sri Widyastuti, Ni Wayan Riyani Martyasari, Angga Susmana Abidin, Hasriaton Padmi, Ervina Handayani, Ni Wayan Putri Utami, Farreh Alan Maulana, Muhammad Syach Maulad Ichfa, Eka Sunarwidhi Prasedya

**Affiliations:** 1 Department of Pharmacy, Faculty of Medicine, University of Mataram, Mataram 83115, Indonesia; 2 Department of Informatics Engineering, Faculty of Engineering, University of Mataram, Mataram 83115, Indonesia; 3 PRTPP, National Research and Innovation Agency (BRIN), Yogyakarta 55861, Indonesia; 4 Faculty of Food Technology and Agroindustry, University of Mataram, Mataram 83115, Indonesia; 5 Bioscience and Biotechnology Research Centre, Faculty of Mathematics and Natural Sciences, University of Mataram, Mataram 83115, Indonesia

**Keywords:** marine, brown algae, lipophilic, antimelanoma, antioxidant activity, untargeted metabolomics, multivariate analysis

## Abstract

Indonesia has high biodiversity of algae that are under-utilised due to limitations in the processing techniques. Here, we observed the effect of two different extraction methods (cold maceration and ultrasonic-assisted extraction (UAE)) on multiple variables of Indonesian brown algae ethanol extracts (*Sargassum polycystum*, *Sargassum cristaefolium*, *Sargassum aquifolium* and *Turbinaria ornata*). The variables observed included metabolites screening by untargeted metabolomics liquid chromatography-high-resolution mass spectrometry (LC-HRMS), observation of total phenolic content (TPC), total flavonoid content (TFC), anti-oxidant and B16-F10 melanoma cells cytotoxicity. UAE extracts had higher extraction yield and TPC, but no TFC difference was observed. UAE extract had more lipophilic compounds, such as fatty acids (Palmitic acid, Oleamide, Palmitoleic acid, Eicosapentaenoic acid, α-Linolenic acid, Arachidonic acid), lipid-derived mediators (11,12-Epoxyeicosatrienoic acid ((±)11(12)-EET)), steroid derivatives (Ergosterol peroxide), lipophilic metabolite (Fucoxanthin), and lipid-soluble vitamins (all-trans-retinol). Principle component analysis (PCA) revealed that TPC, not TFC, in the UAE extracts was correlated with the anti-oxidant activities and cytotoxicity of the extracts towards B16-F10 melanoma cells. This means other non-flavonoid phenolic and lipophilic compounds may have contributed to its bioactivity. These results suggest that out of the two methods investigated, UAE could be a chosen method to extract natural anti-melanogenic agents from brown algae.

## 1. Introduction

The marine habitat has gained wide popularity for study due to its high biodiversity, which provides more than 25,000 potential sources of bioactive metabolites [1]. One known potential marine source is the brown algae. These algae have been studied for various bioactivities, including its use in skin health related to UV exposure. Massive exposure of UV light could cause damage to cellular components that leads to non-melanoma skin cancers, basal cell carcinoma, squamous cell carcinoma, Merkel cell carcinoma, and cutaneous malignant melanoma [2]. Cutaneous melanoma is a highly dangerous skin cancer, which could even lead to mortality. A total of 325,000 melanoma cases were reported in 2020, and 57,000 of the cases have led to death. If this continues, it is estimated that in 2040, there will be a 50% increase in the melanoma cases, with 68% of the cases potentially leading to death [3]. This causes an urgent need for a potential natural anti-melanogenic agent that could possibly come from marine sources, such as brown algae.

*Sargassum serratifoilum*, a brown algae harvested from the coastal areas of Busan, Korea, has been reported to reduce melanin synthesis in α-melanocyte-stimulating hormone (α-MSH)-stimulated B16-F10 melanoma cells [4]. More specifically, other studies have shown that several compounds isolated from brown algae, including dioxinodehydroeckol from *Ecklonia stolonifera* [5] and fucoidan, a lipophilic metabolite, isolated from *Fucus vesiculosus* [6], have anti-melanogenic activity. Another species of brown algae, *Sargassum cristaefolium*, harvested in the western coastal area of Lombok, Indonesia, also has potential skin health effects. It was found to induce the skin healing process after UV radiation, where this algae effectively inhibited proinflammatory TNF-α and IL-6 expression and induced IL-10 production in BALB/c mice skin [7]. A brown algae phenolic compound known as dieckol is also shown to suppress UVB-induced skin damage [8]. Moreover, another study has shown that not only hydrophilic metabolites that has bioactivities, but the lipid extracts from brown algae, such as *Ulva rigida*, *Codium tomentosum*, *Palmaria palmata*, *Gracilaria gracilis*, *Porphyra dioica*, and *Fucus vesiculosus*, also has anti-oxidant activity [9]. There is also growing evidence that interaction between hydrophilic antioxidants and lipophilic antioxidants increases the anti-oxidant capacity of a plant extract, which suggests that ethanol could be a promising extraction solvent. Increased anti-oxidant capacity could also contribute to anti-cancer activity, including anti-melanoma activities.

These brown algae are found in coastal areas in numerous countries, including Indonesia. Lombok, Nusa Tenggara Barat (NTB) is one of the coastal areas of Indonesia that has reported owning numerous marine biotas, including algae [10]. Despite the abundant amount of these algae on the NTB coast, it is still under-utilised by the society or the industry. This is due to the limited information of the processing technique. This increases the need for an efficient extraction method. Choosing an appropriate pre-processing technique of natural products is important. Moreover, algae processing requires a more effective extraction technology, which not only results in a higher yield, but also more potential bioactivity, while also reducing the use of toxic chemicals and generation of waste [11]. It has been reported that various factors, including temperature, pH, and extraction time, significantly affect the yield of fucoidan, an active component of algae [12]. The algae’s alginates are also affected by the manufacturing process [13]. The choice of extraction condition has also been found to affect phlorotannin content in the brown algae, *Silvetia compresa*, where phlorotannin yield was greatly enhanced by ultrasound power density, meanwhile the polysaccharide yield was enhanced when ethanol was presented in a low amount in the solvent [14]. Therefore, in processing these brown algae, choosing an extraction method is a critical step which includes the consideration of various factors such as equipment convenience, cost, extraction efficiency, and time consumption [15]. Here, we describe the effect of using two common extraction methods, cold maceration and ultrasonic-assisted extraction (UAE) for extraction of bioactive compounds from different species of brown algae found in Lombok coast, NTB, Indonesia, which are *Sargassum polycystum*, *Sargassum cristaefolium*, *Sargassum aquifolium* and *Turbinaria ornata*. This method used a safe extraction solvent, ethanol, which could also extract both hydrophilic and lipophilic compounds. Multiple variables were observed, including phytochemical components analysis by untargeted metabolomics, total flavonoid contents and total phenolic contents determination, extraction yield determination, and bioactivity including antioxidant ability and cytotoxic effect towards B16-F10 melanoma cells of the ethanol extracts. A multivariate analysis was conducted to examine the correlation between these variables.

## 2. Results and Discussion

The quantity of secondary metabolites measured are affected by various factors including geographic location, collection time, and extraction protocol [15]. Extraction method optimisation is the most critical and important stage in drug development from natural recourses. Different extraction methods and solvents are normally compared to obtain the most efficient and optimum method, which could provide the highest yield of the active compounds from the solid matrix, which also results in better bioactivity [14,16]. Cold maceration and UAE techniques are both common extraction protocols used in extracting phytochemical components from natural resources. They differ in terms of time needed, technique and equipment used. Cold maceration is more time-consuming than UAE, but it requires less advanced equipment. Meanwhile UAE extraction protocol needs a more advance equipment. In general, UAE is known to be preferable compared to the conventional cold maceration technique. It requires less solvent extraction compared to cold maceration [17,18]. Moreover several other studies have also shown that UAE also results in higher recovery yields of algae metabolites, such as algal lipids [19], algae phenolic compounds [20], and algae carotenoids [21]. It has also been shown to result in 6–35% more of polyphenolic compounds being produced compared to traditional methods [22,23]. Various factors may affect the decision making of choosing the optimum protocol, including the intended pharmacology activity. Therefore, in this study, *Sargassum polycystum*, *Sargassum cristaefolium*, *Sargassum aquifolium* and *Turbinaria ornata*, collected from the Indonesian coast, were extracted with ethanol with two common extraction methods, cold maceration and UAE. Then, multiple variables were observed in order to choose the most optimum method.

### 2.1. Untargeted Metabolomics LC-HRMS Analysis

**Figure 1:**
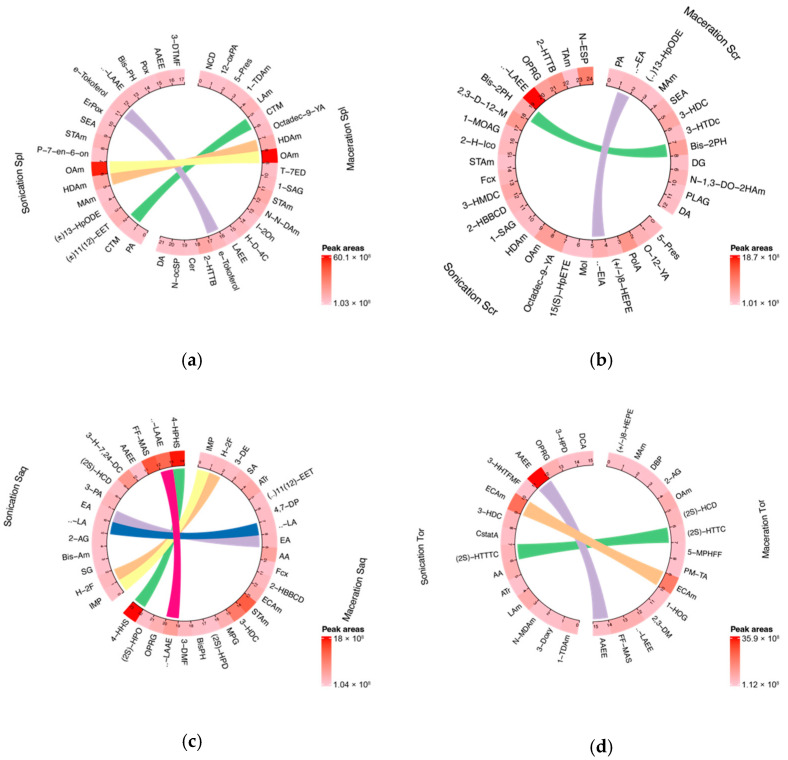
Circular plot showing the variability of chemical compounds compositions in brown algae extracts in two different extraction methods, sonication or ultrasonic-assisted extraction (UAE), and cold maceration. Each plot consists of compounds found in algae that have been extracted with two different methods that are shown side by side on the circular plot. The linear line between each circular plot corresponds to the identic compound found in both type of extraction method. (**a**) *Sargassum polycystum*; (**b**) *Sargassum cristaefolium*; (**c**) *Sargassum aquifolium*; (**d**) *Turbinaria ornata*; Spl = *Sargassum polycystum*; Scr = *Sargassum cristaefolium*; Saq = *Sargassum aquifolium*; Tor = *Turbinaria ornata*. Abbreviations and complete datasets could be seen in Supplementary Materials: Table S1 (a–h).

Due to the complexity of a crude extract, the bioactive compound responsible for its activity could not be concluded straightforwardly. Therefore, in this study, we profiled the chemical compositions of brown algae samples extracted with ethanol and two common extraction technique, UAE and cold maceration (Figure 1). The analysis was performed by using untargeted metabolomics liquid chromatography-high-resolution mass spectrometry (LC-HRMS). This also gave a description of how the extraction technique might affect the phytochemical components of the extract, especially the lipophilic compounds. Figure 1 shows the difference in the variability of the compounds found in four brown algae between two extraction techniques, UAE and cold maceration. Overall, the untargeted metabolomics study revealed that the UAE extracts contained more potential lipophilic compounds compared to the cold macerated extracts. The LC-chromatograms are shown in Supplementary Materials: Figure S1.

#### 2.1.1. *Sargassum polycystum* Ethanol Extract Phytochemical Compounds

The UAE extract of *Sargassum polycystum* contained 17 metabolites. Among the detected species, several are classified as lipophilic, fatty acids and fatty acids-derivative including Palmitic acid (PA), 11,12-Epoxyeicosatrienoic acid ((±)11(12)-EET), Myristamide, Stearamide, Oleamide, Stearoyl-ethanolamide, and γ-Linolenic acid ethyl ester. Fatty acids and its derivatives have been reported to have activity towards melanoma cells [24,25,26]. Meanwhile, Hexadecanamide was also detected in this extract. It is a PA derivate, another form of lipophilic compound. Unfortunately, its role in melanoma cells is not yet understood. Another compound of interest detected in the UAE *Sargassum polycystum* extract was Ergosterol peroxide, a steroid derivate, which has anti-melanogenic activity in the mouse melanoma cell line [27]. Meanwhile the macerated extract of *Sargassum polycystum* contained 21 metabolites, and most of them are also fatty acid derivates. PA was not detected in this extract, however several other fatty acid derivates were detected including 1-1-Tetradecylamine, Lauramide, Hexadecanamide, Stearamide, N,N-Diethyldodecanamide, Ceramide (d18:1/16:0), and N-octodecanoylsphinganine. Ceramides are beneficial to inducing cell apoptosis in melanoma cells, where it induced JNK activation, leading to melanoma cell apoptosis [28]. The pharmacological role of other lipids and lipid derivates presented in the macerated extract of *Sargassum polycystum* are not yet understood.

#### 2.1.2. *Sargassum cristaefolium* Ethanol Extract Phytochemical Compounds

In the UAE *Sargassum cristaefolium* extract, 24 metabolites were detected. As seen in the *Sargassum polycystum* UAE extract, *Sargassum cristaefolium* also contained several lipophilic compounds such as fatty acids and fatty acid derivates, some of which had potential activity on melanoma cells, including Palmitoleic acid [29]; additionally, α-Eleostearic acid, a conjugated fatty acid, also found in the extract, had tumor growth suppression activity via lipid peroxidation in human colon cancer cells [30]. Other than fatty acids, Fucoxanthin, a well-known algal carotenoid and a lipophilic compound, was also detected. Fucoxanthin was able to inhibit the growth of melanoma cell lines and limit melanoma tumour growth [31,32]. Oleamide and γ-Linolenic acid ethyl ester, which both have activity in cancer cells including melanoma cells [33], are also found in UAE *Sargassum cristaefolium* extract. Another compound detected was N-eicosanoylsphinganine, a free form of ceramides. Even though its direct activity in melanoma cells has not been reported, it is part of the ceramides group, which is known to have anti-tumour activity on melanoma cells [28]. Meanwhile in the macerated extract of *Sargassum cristaefolium*, 12 compounds were detected, including Palmitic acid, Myristamide and Stearamide.

#### 2.1.3. *Sargassum aquifolium* Ethanol Extract Phytochemical Compounds

In the UAE *Sargassum aquifolium* extract, 14 metabolites were detected including lipophilic compounds such as Sphinganine, a fatty acid derivative which has been reported to have a role in non-melanoma skin cancer [34]. Eicosapentaenoic acid, also detected, is an unsaturated fatty acid that is known to have potential benefits in UVR-related skin disorders [35,36,37]. Meanwhile, α-Linolenic acid was also detected. It has the most potential anti-melanoma activity compared to PA, linoleic acid and palmitoleic acid in SK-Mel23 cells [38]. No other information was obtained related to anti-melanoma activity of the other detected compounds. In the *Sargassum aquifolium* macerated extract, 23 metabolites were detected which had anti-melanoma activity, including lipophilic compounds such as α-Linolenic acid, Arachidonic acid [38], and Fucoxanthin [31,32].

#### 2.1.4. *Turbinaria ornata* Ethanol Extract Phytochemical Compounds

The UAE and cold macerated *Turbinaria ornata* extract were also analysed. The UAE extract of *Turbinaria ornata* contained 15 metabolites, mainly consisting of fatty acid and its derivates, such as Arachidonic acid and Arachidonic acid ethyl ester. Arachidonic acid has toxicity towards B15-F10 murine melanoma cells [38]. All-trans-retinol was also detected in the UAE *Turbinaria ornata* extract, which is known to have the ability to induce apoptosis in primary and metastatic melanoma cells [39,40,41]. Callystatin A, a polyketide found in this extract, has cytotoxicity on tumour cell line [42]. Meanwhile, the macerated extract of *Turbinaria ornata* contained 15 metabolites. Several fatty acids and fatty acids derivates detected include Oleamide and γ-Linolenic acid ethyl ester. Oleamide [33] has cytotoxicity towards melanoma; meanwhile, γ-Linolenic acid ethyl ester and the other fatty acids detected in this extract have not been reported yet to have anti-melanoma activity.

### 2.2. Determination of Extraction Yield, Total Phenolic Contents (TPC), Total Flavonoid Contents (TFC) of Brown Algae (Sargassum polycystum, Sargassum cristaefolium, Sargassum aquifolium and Turbinaria ornata)

**Figure 2:**
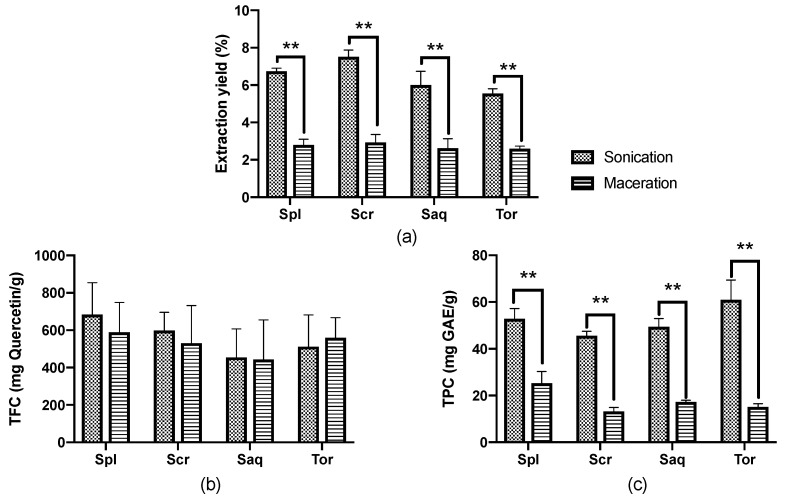
(**a**) Extraction yield, (**b**) Total flavonoid contents (TFC), and (**c**) Total phenolic contents (TPC) from four brown algae; *Sargassum polycystum* (Spl), *Sargassum cristaefolium* (Scr), *Sargassum aquifolium* (Saq) and *Turbinaria ornata* (Tor), extracted with two different extraction techniques, namely cold maceration and sonication, or ultrasonic-assisted extraction (UAE). Data are represented as mean ± SD, and the statistical difference in bioactive compounds for each brown algae are represented as ** *p* ≤ 0.01.

The extraction yield, total phenolic contents (TPC) and total flavonoid contents (TFC) of these extracts were then analysed. As mentioned, the cold maceration used a traditional technique, which required 3 days; meanwhile, the UAE used a much more advanced equipment and only required 90 min. The results showed that the difference in the extraction protocol used had a significant effect on the extraction yield of the four brown algae (*Sargassum polycystum*, *Sargassum cristaefolium*, *Sargassum aquifolium* and *Turbinaria ornata*). In all four brown algae, the extraction yield achieved by UAE was significantly higher compared to the cold maceration extraction. These brown algae extracted with UAE also resulted in a higher TPC compared to the brown algae extracted with the cold maceration technique. However, there was no significant difference in the TFC between both techniques (Figure 2).

Similar results were also shown in the literature where UAE resulted in a higher extraction yield, TPC and even TFC in several other types of brown algae extract, such as *Fucus serratus, Fucus vesiculosus, Fucus spiralis, Himanthalia elongata, Halidrys siliquosa, Laminaria digitata, Laminaria saccharina, Laminaria hyperborea, Ascophyllum nodosum, Alaria esculenta* and *Pelvetia caniculata,* which were harvested from the Irish Sea [43]. The UAE process alone has been able to extract various compounds from macroalgae, including polysaccharides (FSPs, glucans and other antioxidant compounds) [44,45,46]. It is also found to be more efficient in extracting phenolic compounds with a higher molecular weight [46]. Slight differences in the results obtained in the present study compared to the study conducted by Ummat V [43] might be due to the difference in the frequency used: in this study we used 30 kHz, while in the study conducted by Ummat V, 2020, they used 35 kHz. It could also be due to the difference in the species of the samples analysed, as it has also been showed in the previous study that the result of the extraction technique was species-specific [43].

### 2.3. Antioxidant Activity

**Figure 3:**
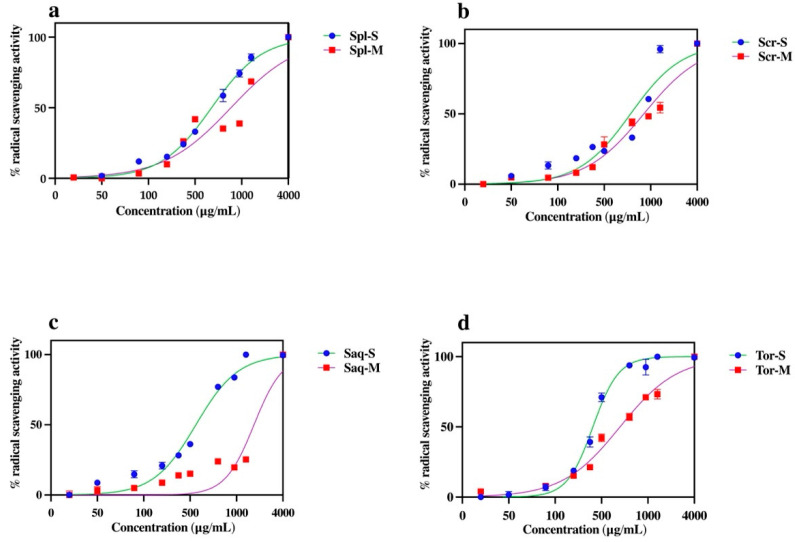
The effect of extraction techniques, ultrasonic-assisted extraction (UAE/sonication) and cold maceration, on the percentage of radical scavenging activity of four brown algae; *Sargassum polycystum* (Spl), *Sargassum cristaefolium* (Scr), *Sargassum aquifolium* (Saq) and *Turbinaria ornata* (Tor). Results are expressed as mean ± SD.

Marine algae have long been known as a rich source of anti-oxidant compounds [47]. Therefore, we here assessed the antioxidant activity of the ethanol extract of four brown algae, *Sargassum polycystum*, *Sargassum cristaefolium*, *Sargassum aquifolium* and *Turbinaria ornata*, by an ABTS method in response to the different extraction protocols used. The results showed that the antioxidant activity of the algae differs in different extraction protocols. The four brown algae (*Sargassum polycystum*, *Sargassum cristaefolium*, *Sargassum aquifolium* and *Turbinaria ornata*), extracted with UAE, had significantly higher antioxidant activity compared to the cold maceration extraction (Figure 3). This significant difference in antioxidant activity was most shown in the *Sargassum aquifolium* extract, where the percentage (%) of radical scavenging activity of the cold macerated *Sargassum aquifolium* extract was only 38.02% at the highest concentration, 10,000 μg/mL. Conversely, the UAE *Sargassum aquifolium* extract reached 100% at only 4000 μg/mL, with the IC_50_ value of 987.75 ± 2.74 µg/mL. The IC_50_ values of the other brown algae, *Sargassum polycystum*, *Sargassum cristaefolium*, and *Turbinaria ornata*, were also significantly lower in the UAE extract compared to the cold macerated extract (Table 1).

The UAE had higher antioxidant activity and also more variability and higher amounts of lipophilic compounds compared to the macerated extract, where the phenolic content was also higher. Phenolic compounds have been known for having antioxidant activity, radical scavenging activities, and also have the ability to induce antioxidant enzyme levels [47]. Therefore, we could assume that the high antioxidant activity in the UAE extract was due to its phenolic and lipophilic contents. This was an interesting issue to study further.

### 2.4. B16-F10 Melanoma Cell Cytotoxicity

**Figure 4:**
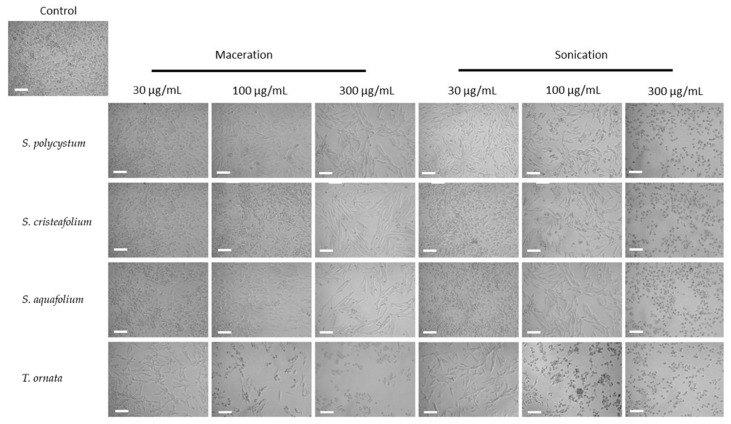
B16-F10 melanoma cell morphology and growth after treatment for 48 h with ultrasonic-assisted extraction (UAE/sonication) and cold maceration extracts of *Sargassum polycystum*, *Sargassum cristaefolium*, *Sargassum aquifolium* and *Turbinaria ornata* at different concentrations (30 μg/mL, 100 μg/mL, and 200 μg/mL); (Scale bar = 100 μM).

**Figure 5:**
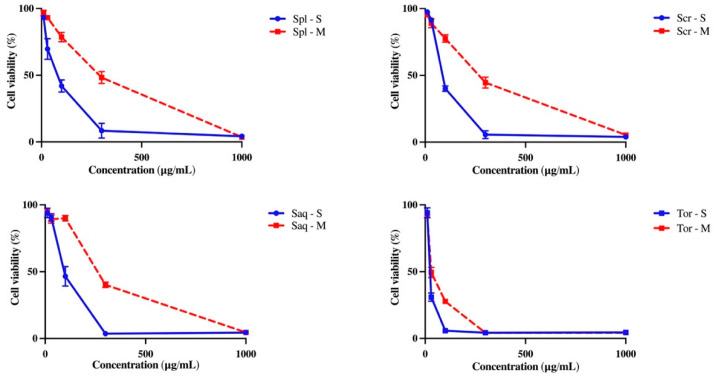
The effect of extraction techniques, cold maceration and ultrasonic-assisted extraction (UAE/sonication) on the cytotoxicity of *Sargassum polycystum* (Spl), *Sargassum cristaefolium* (Scr), *Sargassum aquifolium* (Saq) and *Turbinaria ornata* (Tor) towards the B16-F10 melanoma cell line observed with MTT assay. Results are expressed as mean ± SD.

Antioxidant activity also correlates with various pharmacology activities, including the cytotoxicity towards B16-F10 melanoma cell line, which could also lead to the discovery of a potential anti-melanogenic compound needed to combat skin cancer. Oxidative stress is one of the causes of skin component damage which leads to skin cancer [48]. To assess whether the extraction technique also affected the cytotoxicity towards B16-F10 melanoma cell line of these four brown algae species, B16-F10 melanoma cells were treated with the four brown algae extracts and analysed by an MTT assay. B16-F10 melanoma cell is one of the most used tumor models, which is utilized to understand the mechanisms underlying melanoma progression and also its treatment discovery [49]. Interestingly, the brown algae extracted with the UAE method showed a higher cytotoxic activity towards B16-F10 melanoma cells, compared to the brown algae extracted with the cold maceration technique shown by microscopic observation. At 48 h, it is shown that the cells were less viable after treatment with the four brown algae extracted with UAE, compared to the macerated extracts (Figure 4). MTT assay also showed that the UAE extract was significantly more cytotoxic compared to the macerated extract towards B16-F10 melanoma cell line (Figure 5), as was also shown by a significant difference in the IC_50_ value of both extracts (Table 2).

### 2.5. PCA Analysis

**Figure 6:**
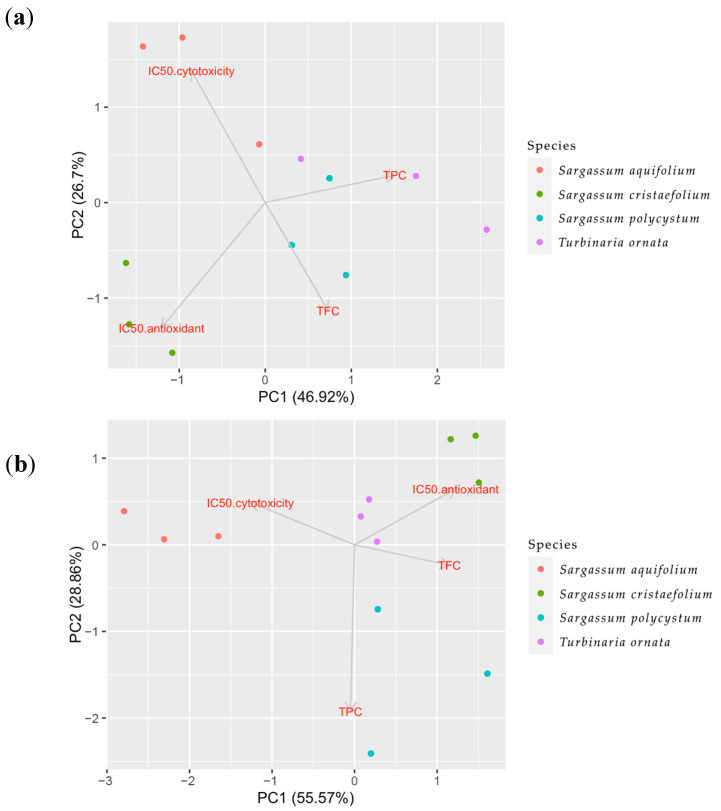
Principle Component Analysis (PCA) of multiple variables, total phenolic contents (TPC), total flavonoid contents (TFC), antioxidant and cytotoxicity towards B16-melanoma cells of the four brown algae species, *Sargassum aquifolium*, *Sargassum cristaefolium*, *Sargassum polycystum* and *Turbinaria ornata*, in two different extraction methods. (**a**) Sonication or ultrasonicated extract (UAE), (**b**) macerated extract.

To further understand the overall influence of the extraction method on total phenolic contents (TPC), total flavonoid contents (TFC), antioxidant effects and cytotoxicity towards B16-F10 melanoma cells, all the data were further analysed using Principal Component Analyses (PCA). In a PCA analysis, the dimensions of the dataset are reduced and the responses were analysed as a result of sample treatment based on the correlation between the data [50]. PCA could also identify the variable that causes the most variation in the dataset [51]. The PC1 is the component that best approximates the data in the least square sense, and the analysis describes the correlation between the variables. Figure 6 shows loading plots with several experimental variables including brown algae species, antioxidant activity, cytotoxicity towards B16-F10 melanoma cell line, and phytochemical compounds (TPC and TFC). PCA is used to see the correlations between the studied variables by using loading plots (Figure 6).

The loading plots describe how vectors may be pinned from the origin point which are PC = 1 and PC = 2. The results could be interpreted by how close the two vectors are. If the vectors form a small angle, this means there is a positive correlation between the two variables. If the vectors form a 90° angle, most likely there is no correlation between the two variables. Meanwhile if the vectors are far from each other and forms a 180° angle, this means the correlation between the two variables are negative.

In the UAE extracts shown in Figure 6a, PC1 (46.92%) and PC2 (26.7%) contribute to the largest variation in the dataset (PC1-PC2 75.62%). Moreover, in Figure 6a, the UAE extracts showed that there was a positive correlation between antioxidant and cytotoxicity, which means the two variables affected each other. Meanwhile, there was a negative correlation between these two variables with the total phenolic contents (TPC). This negative correlation between TPC and IC_50_ of antioxidant and cytotoxicity indicates a positive correlation between TPC and anti-radical scavenging activities and cytotoxicity towards B16-F10 melanoma cell line. This also means TPC affected towards all anti-radical scavenging activities and cytotoxicity towards significantly B16-F10. Furthermore, the TFC variable in this extract showed positive correlation towards the IC_50_ of anti-oxidant activity and cytotoxicity, which means TFC does not affect significantly towards all anti-radical scavenging activities and anti-melanogenic activity.

In the macerated extracts (Figure 6b), this correlation was not shown. Figure 6b shows PC1 (55.57%) and PC2 (28.86%) contribute to the largest variation in the dataset (PC1-PC2 84.43%). There was a positive correlation between TPC and IC_50_ of cytotoxicity, as well as a positive correlation between TFC and IC_50_ of anti-oxidant activity. This means that the TPC does not affect the anti-radical scavenging activities and the TFC does not affect the anti-melanoma activity. On the other hand, a negative correlation was also seen between TPC and IC_50_ of antioxidant activity, and between TFC and cytotoxicity activity, which means TPC affects the anti-radical scavenging activities. Meanwhile, TFC affects the anti-melanoma activity. There was also less correlation between the two activities, the anti-oxidant activity and cytotoxicity towards B16-F10 melanoma cell line.

Analysis of PC1-PC2 75.62% in the UAE extracts also showed that the majority of the *Turbinaria ornata* and *Sargassum aquifolium* (positive area of x axis) were separated from the other two species, *Sargassum cristaefolium* and *Sargassum polycystum*. The analysis also showed that the TFC amount had no effect on the other variables. The PCA analysis on the UAE extracts also showed that *Turbinaria ornata* had the highest TPC amount and has the most antioxidant activity and cytotoxicity towards B16-F10 melanoma cells compared to the other species (Figure 6). In the macerated extracts, PC1-PC2 84.43% showed that TPC and TFC had no effect on either antioxidant or the cytotoxic activity. Furthermore, as shown in the UAE extracts, the macerated extract also showed that the majority of the *Turbinaria ornata* and *Sargassum aquifolium* were separated from the other two species, *Sargassum cristaefolium* and *Sargassum polycystum*. This could also mean that the species coupled together share the same nature. Considering that the TFC did not differ significantly between the two methods (Figure 2), and how the PCA analyses have shown that TFC did not directly affect the antioxidant activity nor the cytotoxicity towards B16-F10 melanoma cells, we assume that the antioxidant or cytotoxic compound might not be a flavonoid group, but rather that it belongs to other compound groups in the extract which are non-flavonoid phenolic and lipophilic compounds.

## 3. Materials and Methods

### 3.1. Collection and Extraction of the Brown Algae

Brown algae samples (*Sargassum polycystum*, *Sargassum cristaefolium*, *Sargassum aquifolium* and *Turbinaria ornata*) were collected at Lendang Luar, North Lombok, Indonesia (8°27′23.1″ S 116°02′07.7″ E). Collected samples were identified with reference to algae electronic database [52]. Upon collection and before transportation to the Laboratorium, brown algae were rinsed with freshwater to remove any sand debris. Clean brown algae were then air-dried and ground into a fine powder before used for extraction. The dried and powdered algae were kept in an air-tight glass container in room temperature until extraction.

### 3.2. Extraction of the Brown Algae

Maceration was conducted as follows: 40 g of dried and powdered brown algae samples were macerated with 400 mL (1:10) of ethanol 96% for 24 h, and this process was repeated three times. At each 24 h, the solution was mixed at every hour only at the first 6 h and then left for 18 h. The solution was then filtered every 24 h with mori cloth. The filtrates obtained from all three repetitions were collected, and then the solvent was evaporated with the vacuum rotary evaporator. Thick filtrates obtained from this process were then used for further experiments. Meanwhile ultrasonic-assisted extraction (UAE) was conducted as follows: 40 g of dried and powdered brown algae samples were soaked in 400 mL of ethanol 96%, then extracted with ultrasonicator (Elmasonic S. Elma Schmidbauer GmbH, Singen, Germany) for 30 min at 30 kHz. This extraction process was repeated three times. At each step, filtrates were filtered with mori cloth and accumulated together; this was followed by solvent evaporation with a vacuum rotary evaporator until thick extracts were obtained, which were then used for further experiments.

### 3.3. Evaluation of Phytochemical Constituents of the Brown Algae

#### 3.3.1. Untargeted Metabolomic Analysis by LC-HRMS

Untargeted metabolomic analysis of the brown algae extracts was conducted using a Q Exactive^TM^ High Resolution Accurate Mass LC-MS/MS (Thermo Scientific^TM^, Waltham, MA, USA) attached to a Thermo Scientific^TM^ VanquishTM Flex UHPLC system. The HPLC solvents used were 0.1 percent formic acid in H_2_O MS grade as solvent A, and 0.1 percent formic acid in Acetonitrile MS grade as solvent B. The HPLC system was as follows: a gradient of 5 percent to 90 percent B in 16 min, an isocratic of 90 percent B for 4 min, and an additional 5 min 90 percent to 5 percent B, and a flow rate of 0.3 mL/min. A volume of 5 μL of sample was injected into the system. The separation was then carried out on a 2.6 m Accucore^TM^ Phenyl Hexyl 100 × 2 mm column, with an MS acquisition range of 150 to 1800 m/z. A sheath gas flow rate of 15 was used, and an auxiliary gas flow rate of 5, a spray voltage of 3.6 kV, a capillary temperature of 320 °C, an auxiliary gas heater temperature of 30 °C, and an S-lens RF level of 50 were used in this assay. The resolution was set to 70,000 for the entire MS, with an AGC target of 3 × 10^6^ and a maximum IT of 250 ms. Moreover, the resolution for dd-MS^2^ was set to 17,500, with an AGC target of 1 × 10^5^ and a maximum IT of 60 ms, and the loop count was set to 5; the (N) CE/steeped was 18, 35, 53, with the TopN and isolation window set to 5 and 1.0 mz, respectively. The minimum AGC target 9 × 10^3^ was used for *dd* setting, with an intensity of 1.3 × 10^5^ and a charge exclusion of 4–8, >8. The exclude isotope was enabled, and the dynamic exclusion time was set to 10 s. Caffeine was used as a calibrant in the study. The compounds were detected based on the Compound Discoverer Library version 3.2.

#### 3.3.2. Determination of Total Phenolic Content

The total phenolic contents (TPC) of the brown algae samples was determined by using the modified Folin–Ciocalteu colorimetric method [53]. A total 10 mg of Gallic acid (GAE) solution in 10 mL of ethanol (1 mg/mL) was used as the reference standard. A series dilution of GAE (10–500 µg/mL) was then prepared, and approximately 100 µL of the sample (1 mg/mL) was combined and mixed with 0.75 mL of the Folin–Ciocalteu reagent (diluted 10-fold in dH_2_O before use). After incubation in room temperature for 5 min, the mixture was then added with 750 µL sodium carbonate (Na_2_CO_3_), which was followed by 90 min incubation. Absorbance was measured at 725 nm with UV-Vis spectrophotometer. TPC of sample was presented as Gallic acid equivalents in milligrams per 100 g of the extract.

#### 3.3.3. Determination of Total Flavonoid Content

Colorimetric assay as described in [54] was used to analyse the total flavonoid content, where 100 µL of sample was diluted with 4 mL of dH_2_O. A volume of 300 µL of 5% sodium nitrite was then added to this mixture and incubated for 5 min. This was then followed by the addition of 300 µL of 10% aluminium chloride and with 6 min incubation. A volume of 2 mL 1 M sodium hydroxide was then added and, immediately, the mixture was diluted by the addition of 3.3 mL dH_2_O and vortexed. The absorbance was determined at 510 nm, and Quercetin was used as the standard for the calibration curve. The total flavonoid content of the sample was presented as mg quercetin equivalents per gram of sample (mg/g).

### 3.4. ABTS Radical Scavenging Assay for Antioxidant Capacity Analysis

The scavenging activity of the brown algae extracts in this study was measured against ABTS radical cation according to the method of [55] with minor modifications. Fresh ABTS solution was prepared for each assay. The stock solutions were prepared as follows: 7 mM ABTS aqueous solution and 2.4 mM potassium persulfate solution. The working solution was prepared as follows: two stock solutions were mixed in equal quantities, followed by 16 h incubation at room temperature in the dark. The solution was then diluted as follows: 250 µL ABTS was mixed with 12 mL ethanol to obtain an absorbance around 0.700 units at 734 nm. A volume of 1 mL of the sample extracts in various concentrations (10–4000 µg/mL) was mixed with 1 mL ABTS solution, followed by the 7 min incubation. Then, the absorbance was measured at 734 nm with a spectrophotometer. The ABTS scavenging activity was calculated with the equation below:

$$\mathrm{Scavenging}\;\mathrm{effect}\;\left(\%\right)=\left[1-\frac{\left(\mathrm{Abs}\;\mathrm{sample}-\mathrm{Abs}\;\mathrm{blank}\right)}{\mathrm{Abs}\;\mathrm{control}}\right]\times 100\%$$

### 3.5. B16-F10 Melanoma Cell Line Viability Assay

B16-F10 melanoma skin cancer cell lines were cultured in Dulbecco’s modified EaGLE medium (DMEM, Wako), supplemented with 10% fetal bovine serum (FBS) at 37 °C in a 5% CO_2_ humidified incubator. Plated cells were then incubated overnight in DMEM, then incubated in DMEM supplemented with specific concentrations of the brown algae extract, and the morphology of the cells was observed at 24 h, 48 h and 72 h after treatment. Cell images were taken by inverted microscope Zeiss Axio Observer Z1 (ZEISS, Oberkochen, Germany). Cytotoxicity of the brown algae extracts was analysed by MTT cytotoxic assay (Cao et al. 2018). Cells were cultured in 96-well culture plate overnight. The next day, the cell culture medium was discarded and changed with a new medium containing several concentrations of brown algae extracts (5–200 µg/mL) then incubated for 72 h. After incubation, cells were added with 50 µL MTT reagent and with 50 µL serum-free medium. Plates were then incubated again in 37 °C 5% CO_2_ for 3 h. After 3 h incubation, MTT solvent was added to the wells, and the wells were shaken for 15 min. The absorbance was then measured at 590 nm and cytotoxicity was calculated.

### 3.6. Statistical Analyses

Data were expressed as mean ± standard deviation (SD). Statistical analyses were performed using multiple *t* tests using the GraphPad Prism (version 9.0, San Diego, CA, USA). Data were considered significantly different if *p* value < 0.05. Principle component analysis (PCA) was utilized to analyse the effect of the extraction method on TPC, TFC, antioxidant and anti-melanoma, and also to learn the correlations between these variables. PCA was carried out using R software (R. software Inc., San Francisco, CA, USA). Metabolomic data presentation was produced using R software (R. software Inc., San Francisco, CA, USA).

## 4. Conclusions

Collectively, the results in this study indicate that the purpose of using the brown algae collected from West Nusa Tenggara Coast, Indonesia, including *Sargassum aquifolium*, *Sargassum cristaefolium*, *Sargassum polycystum* and *Turbinaria ornata*, as a potential natural source of anti-melanogenic agents would be better achieved by extracting the bioactive compounds using the UAE method compared to the conventional maceration technique.

The UAE technique resulted in statistically significant higher extraction yield and total phenolic contents (TPC). The untargeted metabolomic study also revealed that the UAE extraction had resulted in more potential lipophilic compounds, such as fatty acids (Palmitic acid, Oleamide, Palmitoleic acid, Eicosapentaenoic acid, α-Linolenic acid, Arachidonic acid), lipid-derived mediators (11,12-Epoxyeicosatrienoic acid ((±)11(12)-EET)), steroid derivatives (Ergosterol peroxide), lipophilic metabolite (Fucoxanthin), and lipid-soluble vitamins (all-trans-retinols). The UAE extracts also had better antioxidant and cytotoxicity activity towards B16-F10 melanoma cells compared to the extracts obtained from the cold maceration technique. Furthermore, PCA revealed that TFC in the brown algae extract did not contribute to the anti-oxidant or the anti-melanogenic activity of the brown algae extract, which means that other non-flavonoid phenolic and lipophilic compounds may have played the anti-melanogenic role provided by these extracts.

## Figures and Tables

**Table 1:** IC_50_ values for ABTS scavenging of four brown algae species.

Sample	IC_50_	
	Sonication	Maceration
Spl	936 ± 1.61 μg/mL ^a^	1025 ± 1.30 μg/mL ^b^
Scr	1873 ± 7.19 μg/mL ^a^	2258 ± 8.90 μg/mL ^b^
Saq	987.75 ± 2.74 μg/mL	*
Tor	737.89 ± 7.82 μg/mL ^a^	913 ± 5.43 μg/mL ^b^

*Sargassum polycystum* (Spl), *Sargassum cristaefolium* (Scr), *Sargassum aquifolium* (Saq) and *Turbinaria ornata* (Tor). Results are expressed as mean ± SD. Values with different upper case letters (^a,b^) are significantly different at *p* < 0.01. (*) Indicates that IC_50_ could not be calculated, as the radical scavenging activity did not reach 50% at the highest concentration.

**Table 2:** IC_50_ values of brown algae cold maceration and UAE (sonication) extract towards the B16-F10 melanoma cell line observed with MTT assay.

Sample	IC_50_	
	Sonication	Maceration
Spl	70.89 ± 1.851 μg/mL ^a^	259.5 ± 2.414 μg/mL ^b^
Scr	84.01 ± 1.924 μg/mL ^a^	237.8 ± 2.376 μg/mL ^b^
Saq	96.73 ± 1.986 μg/mL ^a^	256.9 ± 2.410 μg/mL ^b^
Tor	26.33 ± 1.421 μg/mL ^a^	39.02 ± 1.591 μg/mL ^b^

*Sargassum polycystum* (Spl), *Sargassum cristaefolium* (Scr), *Sargassum aquifolium* (Saq) and *Turbinaria ornata* (Tor). Results are expressed as mean ± SD. Values with different upper case letters (^a,b^) are significantly different at *p* < 0.01.

## Data Availability

The data presented in this study are available on request from the corresponding author.

## Supplementary Materials

The following supporting information can be downloaded at: https://www.mdpi.com/article/10.3390/molecules27217509/s1, Supplementary Figure S1: Chromatogram of *Sargassum polycystum, Sargassum cristaefolium, Sargassum aquifolium and Turbinaria ornata*; Table S1 (a–h). Compounds in *Sargassum polycystum, Sargassum cristaefolium, Sargassum aquifolium and Turbinaria ornata* extracted with maceration and UAE method detected by LC-HRMS.
