# Supplementary material for: Multivariate Analysis Revealed Ultrasonic-Assisted Extraction Improves Anti-Melanoma Activity of Non-Flavonoid Compounds in Indonesian Brown Algae Ethanol Extract
Source: Molecules. 2022 Nov 3;27(21):7509. doi: 10.3390/molecules27217509 (PMC9655947; doi:10.3390/molecules27217509)
Supplement: Supplementary file 1 [file molecules-27-07509-s001.zip › molecules-1954551-supplementary.pdf]

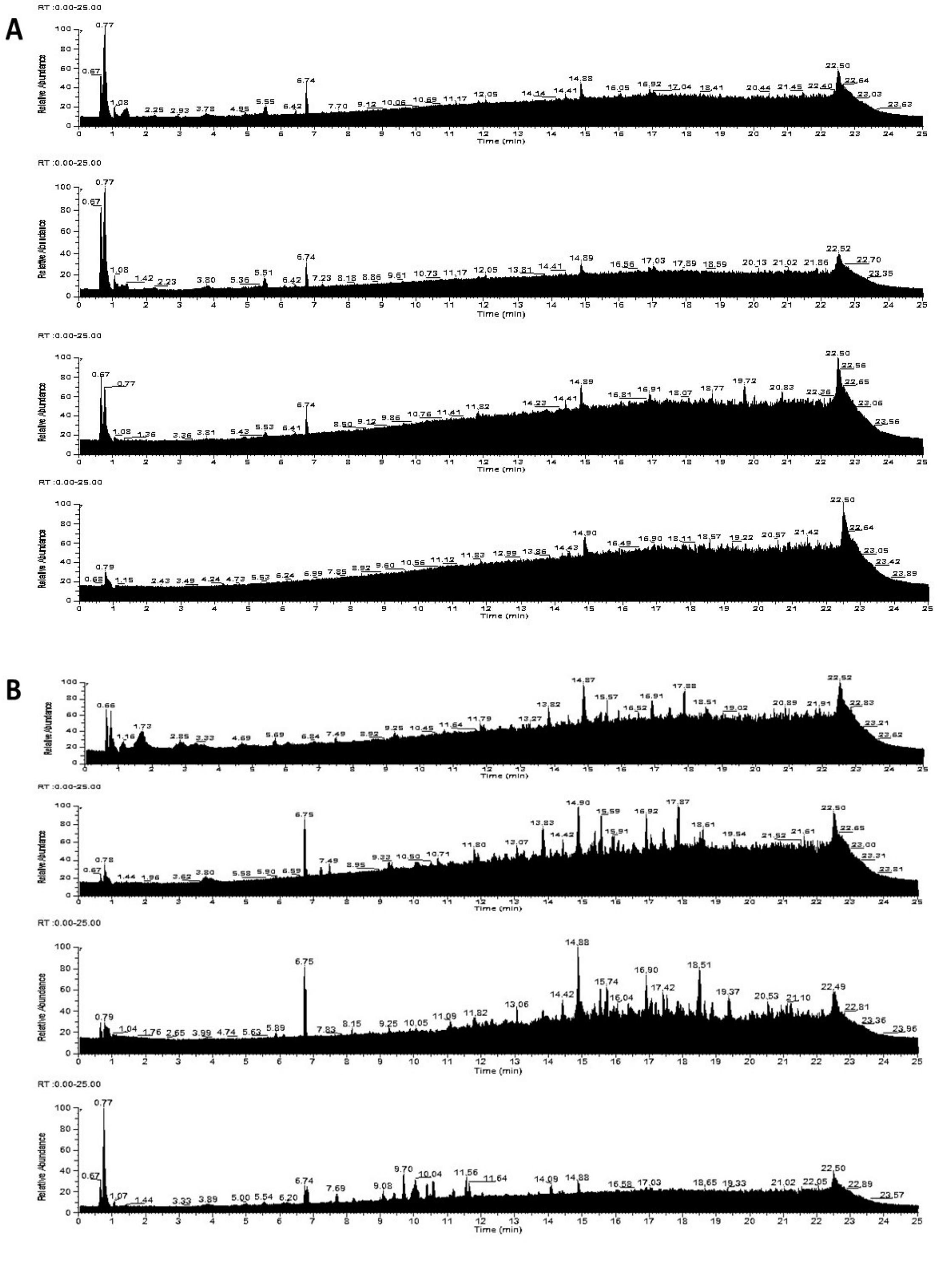

Figure S1. Chromatogram of *Sargassum polycystum, Sargassum cristaefolium*, *Sargassum aquifolium and Turbinaria ornata* (from above to bellow, respectively) extracts using (**A**) maceration (**B**) sonication or UAE

Table S1. (a) Compounds in *Sargassum polycystum* extracted with maceration method detected by LC-HRMS.

Compound	Abbreviation	Formula	RT (min)	Peak Area (intensity)
Nylon cyclic dimer	NCD	C12 H22 N2 O2	5.89	2.25×10^{8}
12-oxo phytodienoic acid	12-oxPA	C18 H28 O3	8.15	1.11×10^{8}
5-Pentylresorcinol	5-Pres	C11 H16 O2	9.25	3.03×10^{8}
1-Tetradecylamine	1-TDAm	C14 H31 N	10.06	2.9×10^{8}
Lauramide	LAm	C12 H25 N O	11.83	6.96×10^{8}
Cetrimonium	CTM	C19 H41 N	12.36	1.13×10^{8}
Octadec-9-ynoic acid	Octadec-9-YA	C18 H32 O2	14.22	3.52×10^{8}
Hexadecanamide	HDAm	C16 H33 N O	14.42	10.9×10^{8}
Oleamide	OAm	C18 H35 N O	14.88	5.72×10^{8}
(2β,5β,9xi,22R)-2,14,22,25- Tetrahydroxycholest-7-ene-3,6-dione	T-7ED	C27 H42 O6	14.96	2.89×10^{8}
1-Stearoylglycerol	1-SAG	C21 H42 O4	15.56	5.31×10^{8}
Stearamide	STAm	C18 H37 N O	15.68	7.71×10^{8}
N,N-Diethyldodecanamide	N-N-DAm	C16 H33 N O	15.89	7.25×10^{8}
Icosaoctaen-2-one	I-2On	C23 H45 N4 O P	16.04	2×10^{8}
(3β,4α,5α)-3- Hydroxyergosta-7,24(28)-diene-4- carbaldehyde	H-D-4C	C29 H46 O2	16.82	5.53×10^{8}
Linolenic acid ethyl ester	LAEE	C20 H34 O2	17.12	1.03×10^{8}
e-Tokoferol	e-Tokoferol	C28 H42 O2	17.19	6.14×10^{8}
2-(3-Hydroxy-3,7,11,15-tetramethylhexadecyl)-3,5,6-trimethyl-1,4-benzoquinone	2-HTTB	C29 H50 O3	18.5	12.8×10^{8}
Ceramide (d18:1/16:0)	Cer	C34 H67 N O3	18.97	1.63×10^{8}
N-octodecanoylsphinganine	N-ocSP	C36 H73 N O3	20.09	2.72×10^{8}
Dichloroacetic acid	DA	C2 H2 Cl2 O2	22.55	1.19×10^{8}

Table S1. (b) Compounds in *Sargassum polycystum* extracted with sonication or UAE method detected by LC-HRMS.

Compound	Abbreviation	Formula	RT (min)	Peak Area (intensity)
Palmitic acid	PA	C16 H32 O2	9.57	1.8×10^8
Cetrimonium	CTM	C19 H41 N	12.65	1.62×10^8
(±)13-HpODE	(±)13-HpODE	C18 H32 O4	13.06	3.64×10^8
(±)11(12)-EET	(±)11(12)-EET	C20 H32 O3	13.06	8.40×10^8
Myristamide	MAm	C14 H29 N O	13.18	1.31×10^8
Hexadecanamide	HDAm	C16 H33 N O	14.41	1.18×10^8
Oleamide	OAm	C18 H35 N O	14.88	60.1×10^8
(3β,5β,20xi,22R)-3,14,20,22,25- Pentahydroxycholest-7-en-6-one	P-7-en-6-on	C27 H44 O6	15.16	1.20×10^8
Stearamide	STAm	C18 H37 N O	15.55	11.7×10^8
Stearoyl ethanolamide	SEA	C20 H41 N O2	15.92	3.02×10^8
Ergosterol peroxide	ErPox	C28 H44 O3	15.95	1.32×10^8
e-Tokoferol	e-Tokoferol	C28 H42 O2	17.2	4.74×10^8
γ-Linolenic acid ethyl ester	γ-LAAE	C20 H34 O2	18.39	4.16×10^8
Bis(3,5,5-trimethylhexyl) phthalate	Bis-PH	C26 H42 O4	18.4	2.00×10^8
Phylloquinone oxide	Pox	C31 H46 O3	18.58	1.13×10^8
Arachidonic acid ethyl ester	AAEE	C22 H36 O2	19.39	4.08×10^8
3-[(17Z)-13,14-Dihydroxy-17-triaconten-1-yl]-5-methyl-2(5H)-furanone	3-DTMF	C21 H38 N6 O3	20.92	1.20×10^8

Table S1. (c) Compounds in *Sargassum cristaefolium* extracted with maceration method detected by LC-HRMS.

Compound	Abbreviation	Formula	RT (min)	Peak Area (intensity)
Palmitic acid	PA	C16 H32 O2	9.56	1.42×10^{8}
α-Eleostearic acid	α-EA	C18 H30 O2	12.75	1.95×10^{8}
(±)13-HpODE	(±)13-HpODE	C18 H32 O4	13.06	1.91×10^{8}
Myristamide	MAm	C14 H29 N O	14.92	1.12×10^{8}
Stearoyl ethanolamide	SEA	C20 H41 N O2	15.92	2.97×10^{8}
(3β,4α,5α)-3-Hydroxyergosta-7,24(28)-diene-4-carbaldehyde	3-HDC	C29 H46 O2	16.39	4.39×10^{8}
(2S)-3-Hydroxy-2-[(9Z)-9-tetradecenoyloxy]propyl (4Z,7Z,10Z,13Z,16Z,19Z)- 4,7,10,13,16,19-docosahexaenoate	3-HTDc	C39 H62 O5	17.52	1.74×10^{8}
Bis-(2-ethylhexyl)-phthalate	Bis-2PH	C24 H38 O4	17.86	5.55×10^{8}
DG-(16:0/18:3(9Z,12Z,15Z)/0:0)	DG	C37 H66 O5	18.46	1.5×10^{8}
N-[(4Z,8Z)-1,3-Dihydroxy-4,8- octadecadien-2-yl]-hexadecanamide	N-1,3-DO-2HAm	C34 H65 N O3	18.66	3.02×10^{8}
1-Palmitoyl-2-linoleoyl-sn-glycerol	PLAG	C37 H68 O5	18.88	1.34×10^{8}
Dichloroacetic acid	DA	C2 H2 Cl2 O2	22.54	1.82×10^{8}

Table S1. (d) Compounds in *Sargassum cristaefolium* extracted with sonication or UAE method detected by LC-HRMS.

Compound	Abbreviation	Formula	RT (min)	Peak Area (intensity)
5-Pentylresorcinol	5-Pres	C11 H16 O2	9.24	2.36×10^{8}
(10Z,14E,16E)-10,14,16-octadecatrien-12-ynoic acid	O-12-YA	C18 H26 O2	11.93	3.06×10^{8}
Palmitoleic acid	PolA	C16 H30 O2	12.29	6.76×10^{8}
(+/-)8-HEPE	(+/-)8-HEPE	C20 H30 O3	12.64	2.51×10^{8}
α-Eleostearic acid	α-ElA	C18 H30 O2	12.74	3.73×10^{8}
Monoolein	Mol	C21 H40 O4	13.08	1.01×10^{8}
15(S)-HpETE	15(S)-HpETE	C20 H32 O4	13.34	1.09×10^{8}
Octadec-9-ynoic acid	Octadec-9-YA	C18 H32 O2	15.51	5.43×10^{8}
Oleamide	OAm	C18 H35 N O	15.52	5.7×10^{8}
Hexadecanamide	HDAm	C16 H33 N O	15.68	1.42×10^{8}
1-Stearoylglycerol	1-SAG	C21 H42 O4	15.74	1.84×10^{8}
(2S)-2-Hydroxy-β,β-carotene-4,4'-dione	2-HBBCD	C40 H52 O3	16.39	2.65×10^{8}
3-Hydroxy-4-methylcholesta-8,24-diene-4-carbaldehyde	3-HMDC	C29 H46 O2	16.45	4.3×10^{8}
Fucoxanthin	Fcx	C42 H58 O6	17.03	2.48×10^{8}
Stearamide	STAm	C18 H37 N O	17.06	1.09×10^{8}
2-Hydroxy-3-(tetradecanoyloxy)-propyl-(5Z,8Z,11Z)-5,8,11-icosatrienoate	2-H-Ico	C37 H66 O5	17.27	2.39×10^{8}
1-myristoyl-2-oleoyl-sn-glycerol	1-MOAG	C35 H66 O5	16.39	2.02×10^{8}
2,3-dihydroxypropyl 12- methyltridecanoate	2,3-D-12-M	C17 H34 O4	17.32	6.33×10^{8}
Bis(2-ethylhexyl) phthalate	Bis-2PH	C24 H38 O4	17.86	3.56×10^{8}
γ-Linolenic acid ethyl ester	γ-LAEE	C20 H34 O2	17.98	18.7×10^{8}
1-Oleoyl-3-palmitoyl-rac-glycerol	OPRG	C37 H70 O5	18.28	5.53×10^{8}
2-(3-Hydroxy-3,7,11,15-tetramethylhexadecyl)-3,5,6-trimethyl-1,4-benzoquinone	2-HTTB	C29 H50 O3	18.5	7.2×10^{8}
Trilaurylamine	TAm	C36 H75 N	18.65	1.08×10^{8}
N-eicosanoylsphinganine	N-ESP	C38 H77 N O3	21.22	9.46×10^{8}

Table S1. (e) Compounds in *Sargassum aquifolium* extracted with maceration method detected by LC-HRMS.

Compound	Abbreviation	Formula	RT (min)	Peak Area (intensity)
Isometheptene	IMP	C9 H19 N	0.8	1.4×10^8
Hexyl 2-furoate	H-2F	C11 H16 O3	6.12	2.1×10^8
3-deoxyestrone	3-DE	C18 H22 O	11.09	1.6×10^8
Stearidonic acid	SA	C18 H28 O2	12.31	3×10^8
All trans retinal (Retinols)	ATr	C20 H28 O	13.07	4.1×10^8
(±)11(12)-EET	(±)11(12)-EET	C20 H32 O3	13.27	1.4×10^8
4,7-diphenyl[1,10]phenanthroline	4,7-DP	C24 H16 N2	14.14	1.7×10^8
α-Linolenic acid	α-LA	C18 H30 O2	14.73	1.5×10^8
Eicosapentaenoic acid	EA	C20 H30 O2	14.83	1.6×10^8
Arachidonic acid	AA	C20 H32 O2	15.37	4.9×10^8
Fucoxanthin	Fcx	C42 H58 O6	16.44	1.1×10^8
(2S)-2-Hydroxy-beta,beta-carotene-4,4'-dione	2-HBBCD	C40 H52 O3	16.46	2.3×10^8
Erucamide	ECAm	C22 H43 N O	17.03	3.8×10^8
Stearamide	STAm	C18 H37 N O	17.04	1.3×10^9
(3beta,4alpha,5alpha)-3- hydroxycholesta-7,24-diene-4-carbaldehyde	3-HDC	C28 H44 O2	17.43	9.7×10^8
1-myristoyl-3-palmitoyl-rac-glycerol	MPG	C33 H64 O5	17.55	3.5×10^8
(2S)-3-Hydroxy-2-(tetradecanoyloxy)propyl-(4Z,7Z,10Z,13Z,16Z,19Z)- 4,7,10,13,16,19-docosahexaenoate	(2S)-HPD	C39 H64 O5	17.57	1.1×10^8
Bis(3,5,5-trimethylhexyl) phthalate	BisPH	C26 H42 O4	17.82	1.4×10^8
3-[(19Z)-15,16-dihydroxy-19-dotriaconten-1-yl]-5-methyl-2(5H)-furanone	3-DMF	C37 H68 O4	18.42	2.3×10^8
γ-Linolenic acid ethyl ester	γ-LAAE	C20 H34 O2	18.5	5.6×10^8
1-Oleoyl-3-Palmitoyl-Rac-Glycerol	OPRG	C37 H70 O5	18.65	3.3×10^8
2S)-1-Hydroxy-3-{[(9Z)-18-hydroxy-9-octadecenoyl]oxy}-2-propanyl (9Z,12Z)-18-hydroxy-9,12-octadecadienoate	(2S)-HPO	C39 H70 O7	18.88	1.4×10^8
4-(2-Hydroxyethyl)phenyl hydrogen sulfate	4-HHS	C8 H10 O5 S	19.39	1.8×10^9

Table S1. (f) Compounds in *Sargassum aquifolium* extracted with sonication or UAE method detected by LC-HRMS.

Compound	Abbreviation	Formula	RT (min)	Peak Area (intensity)
Isometheptene	IMP	C9 H19 N	0.8	1.6 x10^8
Hexyl-2-furoate	H-2F	C11 H16 O3	6.1	2.3 x10^8
Sphinganine	SG	C18 H39 N O2	9.89	1.0 x 10^8
Bis(2-ethylhexyl) amine	Bis-Am	C16 H35 N	11.25	1.1 x 10^8
2-arachidonoylglycerol	2-AG	C23 H38 O4	14.49	1.3 x 10^8
α-Linolenic acid	α-LA	C18 H30 O2	14.72	2 x 10^8
Eicosapentaenoic acid	EA	C20 H30 O2	14.83	2.2 x 10^8
3-[18-(1-Hydroxy-3-methoxy-3-oxopropyl)-3,7,12,17-tetramethyl-8,13-divinyl-2-porphyrinyl]propanoic acid	3-PA	C35 H36 N4 O5	15.07	2.5 x 10^8
(2S)-2-Hydroxy-β,β-carotene-4,4'-dione	(2S)-HCD	C40 H52 O3	16.46	1.5 x 10^8
3β,4α,5α)-3- Hydroxycholesta-7,24-diene-4- carbaldehyde	3-H-7,24-DC	C28 H44 O2	17.43	6.6 x 10^8
Arachidonic acid ethyl ester	AAEE	C22 H36 O2	18.65	1.1 x 10^8
FF-MAS	FF-MAS	C29 H46 O	18.83	1.4 x 10^9
γ-Linolenic acid ethyl ester	γ-LAAE	C20 H34 O2	18.88	1.2 x 10^9
4-(2-Hydroxyethyl)phenyl hydrogen sulfate	4-HPHS	C8 H10 O5 S	22.54	1.7 x 10^9

Table S1. (g) Compounds in *Turbinaria ornata* extracted with maceration method detected by LC-HRMS.

Compound	Abbreviation	Formula	RT (min)	Peak Area (intensity)
(+/-)8-HEPE	(+/-)8-HEPE	C20 H30 O3	12.64	2 x 10^8
Myristamide	MAm	C14 H29 N O	13.18	1 x 10^8
Dibutyl phthalate	DBP	C16 H22 O4	13.83	1 x 10^8
2-Arachidonoyl glycerol	2-AG	C23 H38 O4	14.49	1 x 10^8
Oleamide	OAm	C18 H35 N O	15.52	6 x 10^8
(2S)-2-Hydroxy-β,β-carotene-4,4'-dione	(2S)-HCD	C40 H52 O3	15.75	4 x 10^8
(2S)-2-[(3E,7E,11E)-13-Hydroxy-4,8,12-trimethyl-3,7,11-tridecatrien-1-yl]-2,7,8-trimethyl-6-chromanol	(2S)-HTTC	C28 H42 O3	15.86	2 x 10^8
5-Methyl-3-{2,6,8,10,13-pentahydroxy-13-[5-(1-hydroxytridecyl)tetrahydro-2-furanyl]tridecyl}-2(5H)-furanone	5-MPHFF	C35 H64 O9	16.39	1 x 10^8
PM-Toxin A	PM-TA	C33 H60 O8	16.51	1 x 10^8
Erucamide	ECAm	C22 H43 N O	16.91	2 x 10^8
1-(9Z-hexadecenoyl)-2-(9Z,12Z-octadecadienoyl)-sn-glycerol	1-HOG	C37 H66 O5	17.08	2 x 10^8
2,3-dihydroxypropyl 12- methyltridecanoate	2,3-DM	C17 H34 O4	17.57	2 x 10^8
γ-Linolenic acid ethyl ester	γ-LAEE	C20 H34 O2	18.18	6 x 10^8
FF-MAS	FF-MAS	C29 H46 O	18.83	6 x 10^8
Arachidonic acid ethyl ester	AAEE	C22 H36 O2	19.39	2 x 10^8

Table S1. (h) Compounds in *Turbinaria ornata* extracted with sonication or UAE method detected by LC-HRMS.

Compound	Abbreviation	Formula	RT (min)	Peak Area (intensity)
1-Tetradecylamine	1-TDAm	C14 H31 N	10,09	3.68×10^8
3-Deoxyestrone	3-Doxy	C18 H22 O	11,09	1.9×10^8
N-Methyldioctylamine	N-MDAm	C17 H37 N	11,41	1.48×10^8
Lauramide	LAm	C12 H25 N O	11,82	6.95×10^8
All trans retinal (Retinols)	ATr	C20 H28 O	13,26	1.27×10^8
Arachidonic acid	AA	C20 H32 O2	13.36	5.59×10^8
(2S)-2-[(3E,7E,11E)-13-Hydroxy-4,8,12-trimethyl-3,7,11-tridecatrien-1-yl]-2,7,8-trimethyl-6-chromanol	(2S)-HTTTC	C28 H42 O3	15.86	4.52×10^8
Callystatin A	CstatA	C29 H44 O4	16.02	0.213×10^8
(3β,4α,5α)-3- Hydroxyergosta-7,24(28)-diene-4-carbaldehyde	3-HDC	C29 H46 O2	16.82	4.25×10^8
Erucamide	ECAm	C22 H43 N O	16.91	21.29
3-{13-Hydroxy-13-[5-(1-hydroxypentadecyl)tetrahydro-2-furanyl]tridecyl}-5-methyl-2(5H)- furanone)	3-HHTFMF	C37 H68 O5	17.53	1.14×10^8
Arachidonic acid ethyl ester	AAEE	C22 H36 O2	18,29	35.9×10^8
1-Oleoyl-3-Palmitoyl-Rac-Glycerol	OPRG	C37 H70 O5	19,39	5.79×10^8
(2S)-3-Hydroxy-2-{[9-(3-methyl-5-pentyl-2-furyl)-nonanoyl]-oxy-propyl-11-(3-methyl-5-propyl-2-furyl)-undecanoate	3-HPD	C41 H68 O7	20,12	1.78×10^8
Dichloroacetic acid	DCA	C2 H2 Cl2 O2	22,55	22.08×10^8
